# Circulating biomarkers during treatment in patients with advanced biliary tract cancer receiving cediranib in the UK ABC-03 trial

**DOI:** 10.1038/s41416-018-0132-8

**Published:** 2018-06-21

**Authors:** Alison C. Backen, Andre Lopes, Harpreet Wasan, Daniel H Palmer, Marian Duggan, David Cunningham, Alan Anthoney, Pippa G. Corrie, Srinivasan Madhusudan, Anthony Maraveyas, Paul J. Ross, Justin S. Waters, William P. Steward, Charlotte Rees, Mairéad G. McNamara, Sandy Beare, John A. Bridgewater, Caroline Dive, Juan W. Valle

**Affiliations:** 1 Centre for Cancer Biomarker Sciences, Cancer Research UK Manchester Institute, Manchester, M20 4BX UK; 2 Cancer Research UK & University College London Cancer Trials Center, London, W1T 4TJ UK; 3 Hammersmith Hospital, Imperial College Healthcare Trust, London, W12 0HS UK; 4 Liverpool Experimental Cancer Medicine Centre, University of Liverpool Cancer Research UK Center, Liverpool, L69 3GL UK; 5 The Royal Marsden, London, SW3 6JJ & Surrey SM2 5PT UK; 6 Leeds Cancer Research UK Clinical Center, Leeds, LS2 9JT UK; 7 Cambridge Cancer Centre, Cambridge University Hospitals NHS Foundation Trust, Cambridge, CB2 0QQ UK; 8 Division of Cancer & Stem Cells, University of Nottingham, Nottingham University Hospitals, Nottingham, NG7 2UH UK; 9 Castle Hill Hospital, Hull, HU16 5JQ UK; 10 Department of Oncology, King’s College Hospital, London, SE5 9RS UK; 11 Kent Oncology Center, Maidstone, ME16 9QQ UK; 12 Leicester Royal Infirmary, Leicester, LE1 5WW UK; 13 University Hospital Southampton NHS Foundation Trust, Southampton, SO16 6YD UK; 14 Hampshire Hospitals NHS Foundation Trust, Basingstoke, RG24 9NA UK; 15 Division of Cancer Sciences, University of Manchester, Manchester, M13 9PL UK; 16 The Christie NHS Foundation Trust, Manchester, M20 4BX UK; 17 UCL Cancer Institute, London, WC1E 6DD UK

**Keywords:** Bile duct cancer, Gall bladder cancer

## Abstract

**Background:**

Advanced biliary tract cancer (ABC) has a poor prognosis. Cediranib, in addition to cisplatin/gemcitabine [CisGem], improved the response rate, but did not improve the progression-free survival (PFS) in the ABC-03 study. Minimally invasive biomarkers predictive of cediranib benefit may improve patient outcomes.

**Methods:**

Changes in 15 circulating plasma angiogenesis or inflammatory-related proteins and cytokeratin-18 (CK18), measured at baseline and during therapy until disease progression, were correlated with overall survival (OS) using time-varying covariate Cox models (TVC).

**Results:**

Samples were available from *n* = 117/124 (94%) patients. Circulating Ang1&2, FGFb, PDGFbb, VEGFC, VEGFR1 and CK18 decreased as a result of the therapy, independent of treatment with cediranib. Circulating VEGFR2 and Tie2 were preferentially reduced by cediranib. Patients with increasing levels of VEGFA at any time had a worse PFS and OS; this detrimental effect was attenuated in patients receiving cediranib. TVC analysis revealed CK18 and VEGFR2 increases correlated with poorer OS in all patients (*P* < 0.001 and *P* = 0.02, respectively).

**Conclusions:**

Rising circulating VEGFA levels in patients with ABC, treated with CisGem, are associated with worse PFS and OS, not seen in patients receiving cediranib. Rising levels of markers of tumour burden (CK18) and potential resistance (VEGFR2) are associated with worse outcomes and warrant validation.

## Introduction

Novel therapeutic options, based on an improved understanding of underlying biology and response to therapy, are urgently needed for patients presenting with advanced biliary tract cancer (ABC). Whilst uncommon in the developed world, biliary tract cancer (BTC including cholangiocarcinoma [CCA], gallbladder and ampullary carcinoma) represent a significant global problem due to areas of high incidence, for instance of liver fluke-associated cholangiocarcinoma in Northern Thailand^1^ and of gallbladder cancer in Chile and India.^2^

Surgery is the cornerstone of curative therapy for BTC; the use of adjuvant therapy has historically been based on meta-analyses of non-randomised series and prospective studies.^3^ The recently presented phase III, randomised, BilCap study has demonstrated an overall survival (OS) benefit from the use of adjuvant use of oral capecitabine following surgery versus surgery alone.^4^ Unfortunately, most patients are present with advanced (non-resectable or metastatic) disease and their survival is ≤3 months, with best supportive care alone.^5^ In the ABC-02 study,^6^ the combination chemotherapy with cisplatin and gemcitabine achieved a median survival of 11.7 months, compared to gemcitabine monotherapy (8.0 months; HR = 0.64, 95% confidence interval (CI) 0.52–0.80; *P* < 0.001), findings which were confirmed in the Japanese BT22 study.^7^ Although this is the international reference regimen,^8^ there is a pressing need to improve the efficacy, given these modest outcomes.

Angiogenesis is one of the hallmarks of neoplasia; the expression of vascular endothelial growth factor (VEGF) is associated with adverse clinical features including the presence of liver metastases in intra-hepatic cholangiocarcinoma (iCCA)^9^ and increased microvessel density (MVD) in both gallbladder cancer^10^ and CCA.^11^ In patients undergoing curative resection, MVD has been identified as an independent prognostic risk factor for OS in lymph node-negative iCCA^12^ and gallbladder cancer,^13^ as well as for disease-free survival (DFS)^13^ and OS^14^ in patients with extrahepatic cholangiocarcinoma (eCCA). These clinical observations are consistent with the demonstration of receptors for VEGF (VEGFR1 and VEGFR2) in tumour proximal endothelial cells^15^ along with the frequent (40–75%) expression of VEGF (particularly VEGFA) in BTC 9-,^11^ particularly at the invasive edge of the tumour.^13^

Cediranib is an oral VEGFR1, VEGFR2 and VEGFR3 tyrosine kinase inhibitor (TKI), with additional activity against platelet-derived growth factor (PDGF) receptors and c-KIT.^16^ In the prospective randomised double blind placebo-controlled phase II ABC-03 study, the cisplatin and gemcitabine combination was evaluated with either cediranib or placebo. Although an improved response rate was observed (44% vs. 19% with placebo; *P* = 0.0036) along with an improved 6-month progression-free survival (PFS, 70.5% vs. 61.3%; *P* < 0.05) in cediranib-treated patients, the magnitude of this effect did not reach the pre-defined level of statistical significance (hazard ratio [HR] for PFS: 0.93, 80% CI 0.74–1.19; *P* = 0.72) for the primary endpoint. This may have been due to lack of efficacy, or alternatively, underpowering of the statistical plan, or because cediranib was not well tolerated in this combination.^17^

Recognising the challenge of serial tumour biopsy, an exploratory translational endpoint of the ABC-03 study was the prospective longitudinal profiling of circulating biomarkers associated with angiogenes is. We now present the findings of this work, set the findings in context and evaluate the implications for future clinical trials.

## Materials and methods

### Patients and treatment

ABC-03 (clinicaltrials.gov NCT0939848) was an investigator-initiated, multi-centre (15 UK sites), double-blind, placebo-controlled, randomised phase II study of cediranib added to the standard-of-care chemotherapy regimen (cisplatin and gemcitabine), the details of which have been described previously.^17^ Permission for this trial was granted by the North West 5 Research Ethics Committee, Haydock Park on 23 August 2010 (10/H1010/42). All patients provided written informed consent before randomisation.

### Material collection and analysis

Blood samples were collected from the patients for biomarker studies into EDTA tubes and processed into plasma at up to 11 timepoints; two pre-treatment baseline samples and then on the first day of cycles 2–8, at the end of chemotherapy and 1-month after the end of chemotherapy. The circulating markers of angiogenesis (VEGFA, VEGFC, VEGFR1, VEGFR2, angiopoietins 1 and 2 [Ang1, Ang2], fibroblast growth factor b [FGFb], hepatocyte growth factor [HGF], PDGFbb, keratinocyte growth factor [KGF], placental growth factor [PlGF], tyrosine kinase with Ig and EGF homology domains 2 [Tie2], stromal-derived growth factor 1b [SDF1b]) and inflammation (interleukin 6 and interleukin 8 [IL6 and IL8]) were measured with a validated^18^ multiplex enzyme-linked immune-sorbent assay [ELISA] platform (Aushon BioSystems, Billerica, Massachusetts, USA), according to the Good Clinical Practice (GCP) standards at the Cancer Research UK Manchester Institute (Manchester, UK). Concentrations of the circulating total cytokeratin18 (CK18),^19^ released from epithelial cells during death (apoptosis and necrosis), were measured with an M65 ELISA (Peviva, Nacka, Sweden), also previously validated and implemented to GCP as previously described.^20^

Whole-blood (10 mL) was collected in CellSave Preservative Tubes at up to four time points (pre-treatment baseline sample, on day 1 of cycles two and five, and 1-month after the end of chemotherapy) for the enumeration of circulating tumour cells (CTCs) with the CellSearch platform (Janssen Diagnostics, South Raritan, New Jersey, USA) within 4 days of blood draw.^21^ Briefly, after immunomagnetic capture of EpCAM-positive cells, immunophenotyping of cells with an intact (4′,6-diamidino-2-phenylindole [DAPI] stained) nucleus using antibodies cytokeratin (CK) and CD45 allowed the classification of circulating tumour cells as EpCAM^+^, CK^+^, DAPI^+^ and CD45^−^.

All collected samples were analysed, unless the samples were not available for clinical reasons or patient discontinuation from the study (per protocol).

### Statistical methods

Two aliquots of each plasma sample were analysed to determine the biomarker levels. The mean was calculated and used in statistical analyses. Two pre-treatment baseline samples (collected on separate days) were used to establish a mean pre-treatment value. This concentration was assigned to the date that patient was randomised in the trial and used as a reference point to compare with the longitudinal sampling data. So as to retain as much data as possible for analysis, samples which were analysed and found to be above the upper limit of assay detection (ULOD) were assigned a numerical value of 1 pg/mL above the ULOD. Similarly, measurements which fell below the lower limit of assay detection (LLOD) were assigned a numerical value of half of the assay LLOD.

In order to explore the ability of the biomarkers to predict OS based on greatest change from baseline, the percentage change from baseline was calculated. Patients were ranked in order and divided into three groups (tertiles) for comparison. The middle tertile was set to 0, as this represented the ‘least change’ group. The two extremes were compared with this.

The longitudinal sampling data were analysed using time-varying covariate Cox models (TVC), this is a model that considers the proportionality of hazards at any point in time. The HR is obtained by integrating the longitudinal sampling data. All statistical analysis was carried out at the Cancer Research UK, and University College London (UCL) Cancer Trials Centre, London.

The means and 95% CI for each marker were plotted over time by the treatment group to assess the change over time and the difference between the treatment groups. In order to assess whether a change in the marker at 3 months (the time point at which the efficacy evaluation took place) is associated with survival outcomes, patients were grouped in terms of their percentage change at 3 months, from baseline, into three groups based on the distribution of the data (tertiles): lower, mid and higher groups. The mid tertile group was used as the reference group and represented the group of patients with the least percentage change at 3 months from baseline. The lower tertile group included the group of patients with a percentage change decrease at 3 months from baseline. The higher tertile group included the group of patients with a percentage increase at 3 months from baseline. For CTC count, a different approach was used by grouping patients into no detectable CTCs at baseline and at cycle 3 or any detectable CTCs at baseline and at cycle 3. These groups were also compared using standard Cox model for PFS and OS. Considering that the biomarkers were evaluated at different time points and were variable over time, and that the aim of this study was to evaluate the effect of the changing biomarkers over time on the time-to-event outcomes, a TVC approach was performed. The time-to-event endpoints considered were PFS and OS. The TVC models were fitted separately for each biomarker at a time adjusting for treatment. Also, TVC models were fitted separately for each biomarker and the interaction between the treatment and the marker were evaluated.

Considering that there were multiple biomarkers, backward selection was applied to a Cox model including all biomarkers to identify a model with fewer, but relevant variables (parsimonious multivariable Cox model), using a 5%-significance level as a criteria for the selection of a variable into the model. This was done for PFS and OS Cox models with baseline markers (standard Cox model) and TVC. All these models were adjusted for the treatment group, which was considered a fixed variable in the backward selection procedure.

## Results

### Patient information

A total of 124 patients (62 each in the cediranib and placebo groups) with a median age of 65.1 years were recruited between 05 April 2011 and 28 Sept 2012. Details of the patient population have been described previously.^16^ In summary, 104 (84%) patients had metastatic disease (the remainder had locally-advanced disease). The primary disease site was cholangiocarcinoma in 77 patients (62%), gallbladder cancer in 39 (31%) and ampulla of Vater in 8 (6%). The median PFS was 8 months (95% CI 6.5–9.3) in the cediranib group and 7.4 months (5.7–8.5) in the placebo group (HR 0.93, 80% CI 0.74–1.19, *P* = 0.72). The median OS was 14.1 months (95% CI 10.2–16.4) in the cediranib group and 11.9 months (9.2–14.3) in the placebo group (HR 0.86, 80% CI 0.58–1.27, *P* = 0.44).

### Dynamic biomarker changes in response to chemotherapy and cediranib

**Fig. 1:**
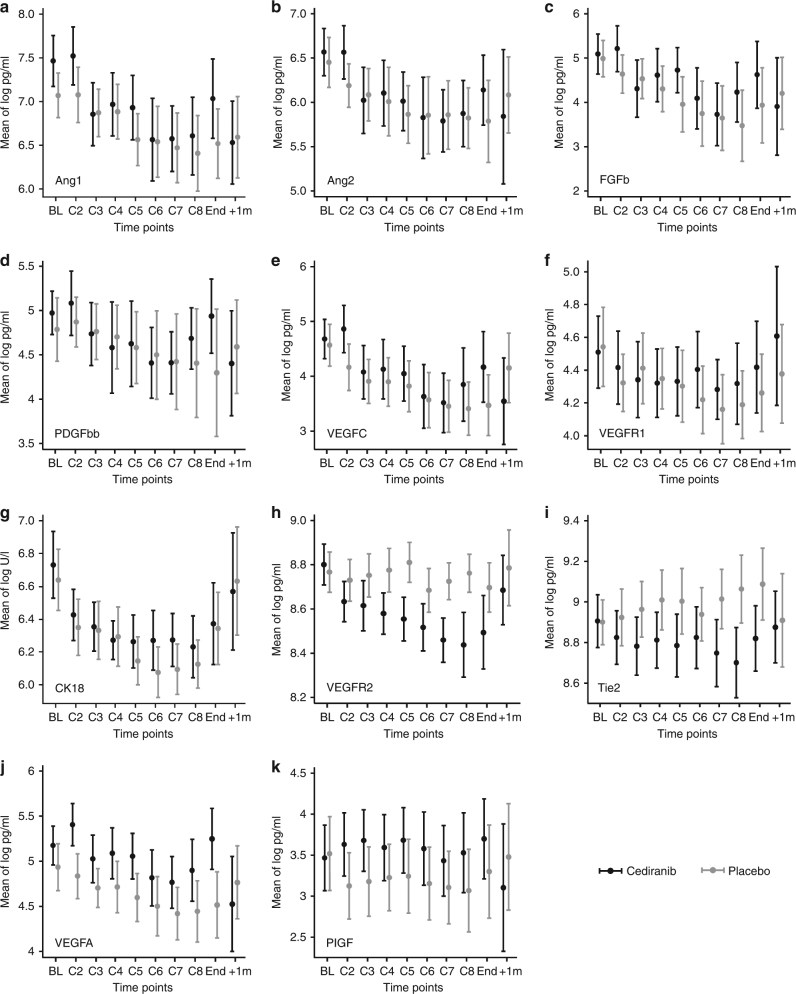
Changes in key biomarkers during treatment, split by treatment arm. The mean log of pg/ml of each biomarker, by treatment arm, is shown at baseline (BL), during treatment cycles (C2-8), at the end of chemotherapy (End) and 1 month after the end of all treatment (+1m) which equates to 1 month after disease progression has been documented. Panels **a**-**g** indicate markers that change similarly in both arms; the cause may be CisGem chemotherapy or tumour burden (rather than Cediranib). Panels **h** and **i** show markers that occur at lower levels in the circulation as a result of treatment with Cediranib. Panels **j** and **k** show markers that appear to be shed into the circulation as a result of Cediranib. Error bars indicate 95% confidence intervals. Number of patients at each time-point; BL=114, C2=96, C3=92, C4=90, C5=79, C6=73, C7=71, C8=59, End=55, +1m=44

Figure 1 describes the changing levels of the multiple biomarkers in each arm over time and demonstrates that there were some differences between the treatment groups. Panels a–g demonstrate a decrease in circulating Ang1 and 2, FGFb, PDGFbb, VEGFC, VEGFR1 and CK18 from the second time-point (prior to cycle 2, i.e. post-cycle 1 of systemic treatment) and that this effect was lost at the time-of-disease progression. This was independent of cediranib, and is likely to be related to chemotherapy and/or disease load. In contrast, panels h and i demonstrate a differential effect of cediranib, preferentially reducing VEGFR2 and Tie2. This difference was again lost at disease progression. Panels j and k demonstrate a reverse effect, whereby cediranib was associated with the preserved levels of circulating VEGFA and PIGF. The effect-neutral biomarkers measured are shown in Supplementary Figure S1 (HGF, IL6, IL8, KGF and SDF1b).

It is important to note that complete datasets are available only for patients who were originally benefitting from the treatment, as, in patients whose disease progressed early, provision of further research samples was discontinued.

### Cediranib attenuates the detrimental outcome associated with rising VEGFA

**Fig. 2:**
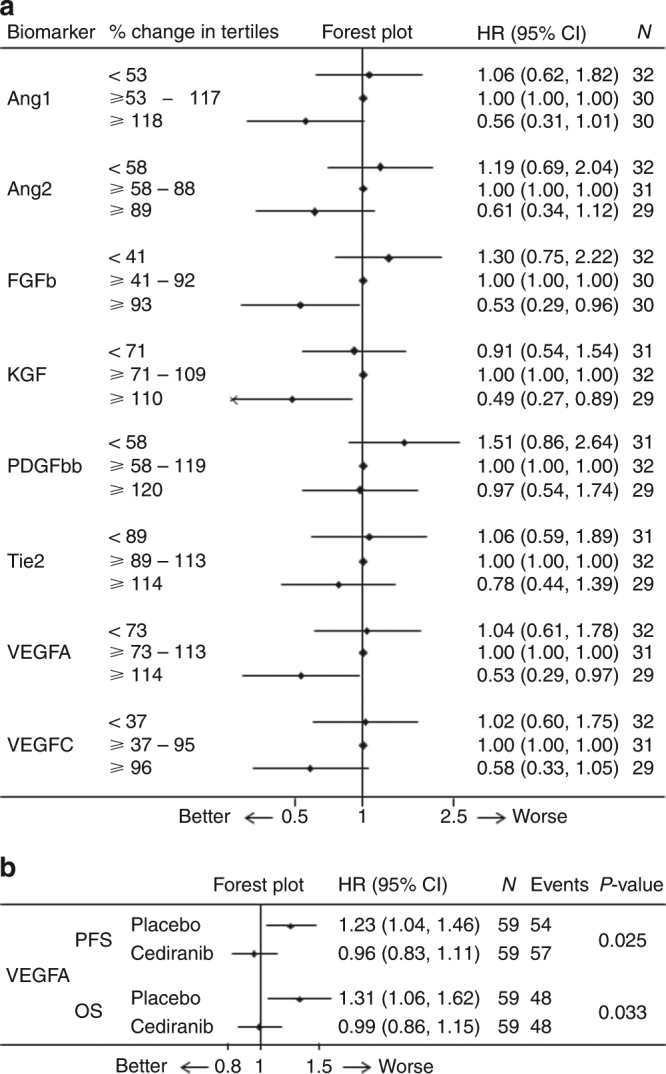
**a** Relationship between % change from baseline at Cycle 3 and overall survival. % change from baseline was calculated, the patients ranked in order and divided into three groups for comparison. The middle tertile was set to 0, as this represents the ‘least change’ group, so the two extremities can be compared with this. **b** Relationship between biomarker change during treatment and outcome. VEGFA change from baseline during treatment was evaluated using the time-varying covariate Cox model, which included VEGFA as a continuous variable and considered units of 100pg/ml change in levels of circulating VEGFA during treatment. At any given time-point, an increase in VEGFA in patients treated with placebo is associated with a shorter PFS and OS. However, an increase in VEGFA in patients treated with cediranib results in a slightly longer PFS and has no effect on OS. The interaction (treatment with biomarker) p-value is shown

Changes at cycle 3 (C3) compared to the baseline for patients in both treatment arms combined (i.e. chemotherapy with placebo and chemotherapy with cediranib) are shown in Fig. 2a. Increased levels of seven biomarkers at C3 describe a group of patients who may benefit from the treatment (with the exception of PDGFbb, in whom, patients with decreased levels at C3 may benefit less from the treatment). The remaining biomarkers measured are shown in Supplementary Figure S2A (CK18, HGF, IL6, IL8, PlGF, SDF1b, VEGFR1 and VEGFR2).

In keeping with the effect seen at cycle 3 (above), patients with increasing levels of VEGFA at any time in the TVC had a worse outcome for both PFS and OS (Fig. 2b). However, in patients who received cediranib, this detrimental outcome was attenuated. All other biomarkers measured are shown in Supplementary Figure S2B.

### Multivariable models for biomarkers predictive of the outcome

Table 1 details multivariable models using the principle of backward selection for biomarkers at baseline and when assessed longitudinally using PFS and OS as outcomes. For the predictive capacity of biomarkers at baseline, two different models are generated for PFS and OS, respectively. These differences are likely to be due to the short-term, rather than the longer-term, biological impact. When considering the change in biomarkers over time, rising levels of Ang2 are associated with longer OS (HR 0.77 [0.64–0.93] *P* = 0.007), but conversely increasing levels of CK18 (a surrogate measure of disease burden/cell death) and VEGFR2 (potentially a mechanism of resistance to therapy) are associated with shorter OS (HR 1.07 [1.04–1.10] *P* < 0.001 and HR 1.12 [1.01–1.23] *P* = 0.02). PDGFbb does not feature in these models as it did when previously described,^16^ primarily because PDGFbb was previously analysed in two subsets (dichotomised at the median), rather than as a continuous variable. The tumour markers CEA, CA19-9 and CA125 are not described, as no longitudinal data was available for them. Supplementary Figure S3 shows the median and the range of all baseline circulating biomarkers by the treatment arm.

**Table 1:** Cox multivariable model at baseline and using time-varying parameters

Biomarker (units)/amount of change considered	Multivariable Cox models using baseline biomarkers				Multivariable models using time-varying biomarkers			
	Progression-free survival		Overall survival		Progression-free survival		Overall survival	
	HR (95% CI)	*p*-value	HR (95% CI)	*p*-value	HR (95% CI)	*p*-value	HR (95% CI)	*p*-value
Ang2 (pg/ml)/1000	—	—	—	—	—	—	0.77 (0.64–0.93)	0.007
CK18 (U/l)/100	1.03 (1.00–1.06)	0.029	1.08 (1.04–1.12)	<0.001	1.05 (1.02–1.09)	0.001	1.07 (1.04–1.10)	<0.001
CTC count/7.5 ml	1.05 (1.01–1.08)	0.01	1.05 (1.02–1.09)	0.004	—	—	—	—
FGFb (pg/ml)/100	0.96 (0.92–1.00)	0.04	—	—	—	—	—	—
HGF (pg/ml)/100	—	—	1.13 (1.05–1.20)	0.001	—	—	—	—
IL6 (pg/ml)/10	—	—	1.04 (1.01–1.07)	0.017	—	—	—	—
IL8 (pg/ml)/10	—	—	0.94 (0.90–0.98)	0.005	—	—	—	—
SDF1b (pg/ml)/100	0.96 (0.93–0.99)	0.014	—	—	—	—	—	—
VEGFC (pg/ml)/100	1.10 (1.01–1.21)	0.038	—	—	—	—	—	—
VEGFR1 (pg/ml)/100	—	—	0.77 (0.66–0.90)	0.001	—	—	—	—
VEGFR2 (pg/ml)/1000	1.16 (1.04–1.3)	0.009	1.15 (1.02–1.29)	0.022	1.14 (1.04–1.24)	0.004	1.12 (1.01–1.23)	0.02
Treatment								
Placebo	1.00	0.142	1.00	0.024	1.00	0.94	1.00	0.93
Cediranib	0.69 (0.42–1.13)		0.57 (0.35–0.93)		1.02 (0.68–1.52)		0.98 (0.63–1.52)	

Each marker was chosen for inclusion using backward selection using significance level criteria of 0.05.The final best PFS Cox model at baseline contained treatment, CK18, CTC count, FGFb, SDF1b, VEGFC and VEGFR2. Increase in all biomarkers (with the exception of FGFb and SDF1b) were associated with an increased risk of disease progression.The final best OS Cox model at baseline contained treatment, CK18, CTC count, HGF, IL6, IL8, VEGFR1 and VEGFR2. Increase in all biomarkers (with the exception of IL8 and VEGFR1) were associated with an increased risk of death.The final best PFS Cox model using time-varying parameters, contained treatment, CK18 and VEGFR2. Increase in both biomarkers (with the exception of FGFb and SDF1b) were associated with an increased risk of disease progression.The final best OS Cox model using time-varying parameters, contained treatment, Ang2, CK18 and VEGFR2. Increase in all biomarkers (with the exception of Ang2) were associated with an increased risk of death

### CellSearch-detected circulating tumour cells are not predictive of the benefit from cediranib

**Fig. 3:**
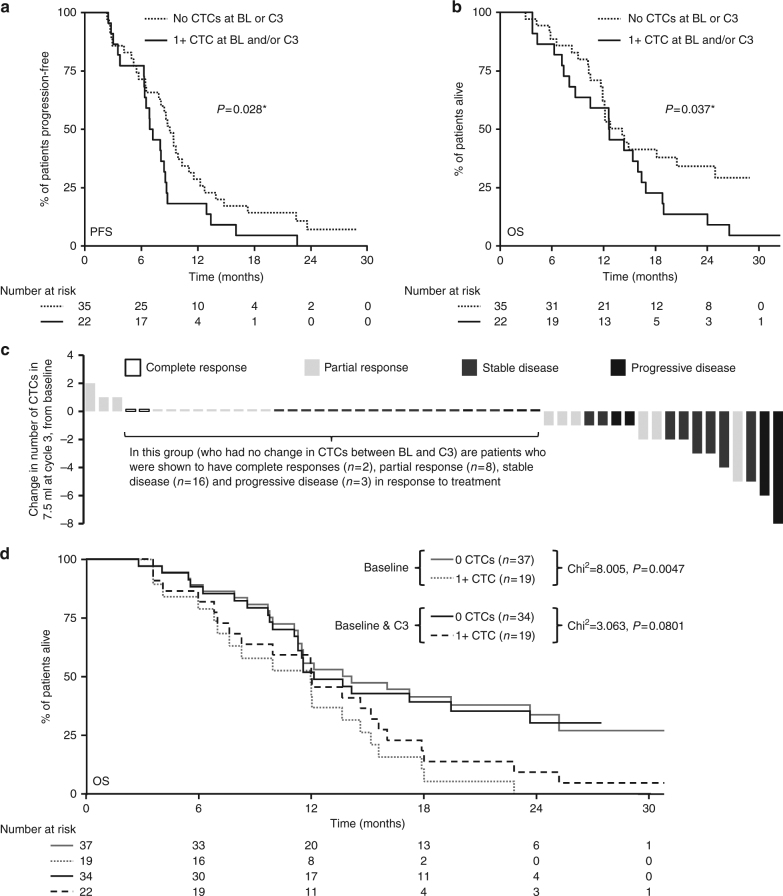
Circulating tumour cells. Association between CTC count at baseline and Cycle 3 and PFS (**a**) and OS (**b**); patients who had CTCs enumerated using Cell Search both at baseline (BL) and at the start of Cycle 3 of treatment, were divided into two categories; Group 1 had no CTCs at BL and C3 (n=35) and Group 2 had at least 1 CTC at either BL or C3, or both time-points (n=22). The range of CTCs observed in this patient set was 0-44, with a median of 0 and a mean of 2 CTCs. *As both of these curves overlap, this p-value may not be reliable. **c** Shows change in CTC count (as absolute numbers at C3), shaded by best response. The hypothesis would be that the patients who had the biggest decrease in CTCs, would have better outcomes (which is not the case). **d** Using the data collected from the n=56 patients who had CTCs enumerated at both baseline and C3, this shows that combining baseline and C3 CTC counts appears less discriminatory than considering baseline CTC counts alone

Changes in CellSearch (CS)-detected CTCs do not predict for patient outcomes for either PFS or OS, as illustrated in Fig. 3. Given the low absolute numbers of CTCs, combined analysis of baseline and cycle 3 CTC numbers did not improve the discrimination over baseline counts alone. As such, assessment of on-treatment CTCs did not predict the outcome.

**Fig. 4:**
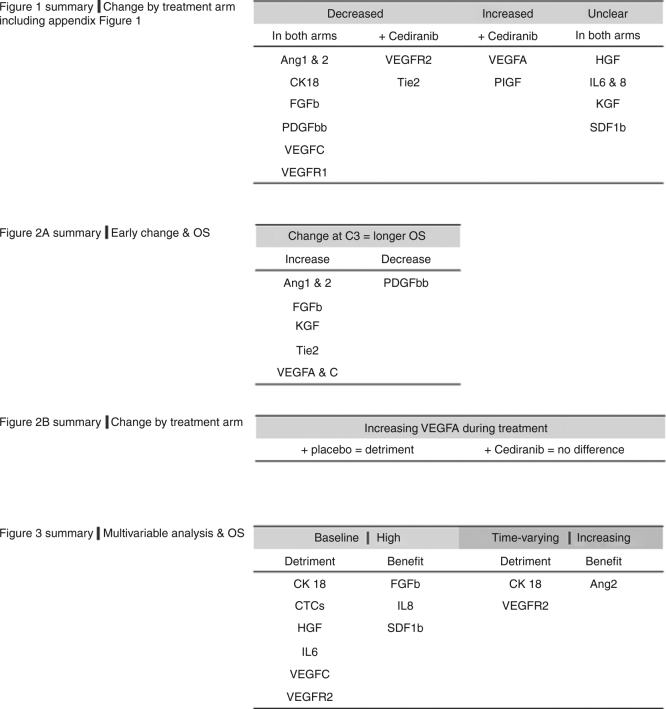
Summary. Figure 1 shows markers which change with treatment, split by treatment arm; Ang1&2, FGFb, PDGFbb, VEGFC and VEGFR1 show patterns of change which are similar in both placebo and cediranib arms. Four proteins show different patterns of change between the two treatment arms; VEGFR2 and Tie2 decrease + Cediranib, VEGFA and PlGF increase + Cediranib. Appendix Figure 1 shows changes with treatment which occurred in HGF, IL6, IL8, KGF and SDF1b, which do not show obvious patterns of change. This information may be relevant when designing future trials. Figure 2 examines how change in biomarkers is related to length of OSIn all patients, increases in Ang1&2, FGFb, KGF, Tie2, VEGFA and VEGFC at C3 was associated with longer OS. Conversely, decrease s in PDGFbb were associated with longer OS. Change in VEGFA is the only marker which predicts outcome (PFS and OS) differently by treatment arm (increasing VEGFA in the placebo arm is associated with a worse outcome, but this not seen in patients in the Cediranib arm). Table 1 summarises univariate and multivariable Cox models using time-varying parameters for PFS and OS

Figure 4 summarises the data presented in Figs. 1–3.

## Discussion

The ABC-03 clinical trial assessed the effect of adding cediranib (an oral VEGFR1, VEGFR2 and VEGFR3 receptor tyrosine kinase inhibitor, with additional activity against PDGF receptors and c-KIT) to cisplatin and gemcitabine chemotherapy in a double-bind, placebo-controlled manner. The study did not meet its primary endpoint (improvement in PFS); however, signals were observed that would support further anti-angiogenesis approaches. Elevated baseline levels of the tumour markers CEA and CA125 (in addition to CA19-9), and total cytokeratin 18 and VEGFR2, as well as CTCs were shown to be prognostic in ABC. Baseline plasma PDGFbb concentrations might predict for the cediranib activity.

The second paper considers the change in circulating biomarkers during the treatment; decrease in circulating Ang1, Ang2, CK18, FGFb, PDGFbb, VEGFC and VEGFR1 was observed in patient samples independent of treatment with cediranib. Cediranib has previously been proposed to be causally linked with a reduction in circulating VEGFR1, both in hepatocellular carcinoma (HCC)^22^ and glioblastoma.^23^ However, both were uncontrolled single-arm studies. In this prospective double-blind placebo-controlled study, we have shown that this observation is not, in fact, due to cediranib, rather due to thechemotherapy or disease load. Similarly, a reduction in circulating plasma Ang2 has been reported in uncontrolled studies in glioblastoma^23^ and HCC.^22^ We demonstrated that this is also independent of cediranib treatment and highlights the importance of a prospective randomised study design in evaluating a potential biomarker.

Our observation of cediranib-induced reduction in VEGFR2 is in keeping with previously published findings and its known mechanism of action. This has been described following cediranib monotherapy in solid tumours; phase I study,^24^ acute myeloid leukaemia,^25^ glioblastoma,^23^ HCC,^22^ gastrointestinal stromal tumour^26^ and in combination with lomustine in glioblastoma,^27^ carboplatin and paclitaxel in cervical cancer^28^ and gefitinib in solid tumours.^29^ Placebo-controlled studies in colorectal cancer,^30^ renal cell cancer,^31^ and breast cancer^32^ confirmed that a reduction in VEGFR2 was due to cediranib and was independent of the companion therapies (primarily a combination with chemotherapy, as in this study). We also observed that cediranib-induced reduction in circulating Tie2 and similar findings have been reported in glioblastoma,^23^ colorectal cancer^30^ and in solid tumours treated with a cediranib–gefitinib combination.^29^

Patients with increasing levels of VEGFA at any time had a worse outcome for both PFS and OS, in patients who received cediranib, this detrimental outcome was attenuated. This, again, is consistent with the known mechanism of action of cediranib.^16^ This suggests that the changes in circulating VEGFA correlates with the potential benefit from treatment with cediranib.

Using multivariable models for biomarkers predictive of outcome, rising circulating levels of Ang2 were shown to be associated with longer OS. Ang2 is a growth factor ligand of the Tie family of protein receptor tyrosine kinases.^33^ Ang2 promotes the dissociation of pericytes and loosens the cellular junctions, which results in unstable blood vessels.^34^ Increasing levels of circulating Ang2 in this setting would appear to be indicative of effective tumour destabilisation.

Conversely, rising levels of CK18 were associated with a shorter OS. Cancers of epithelial origin are known to contain relatively large intracellular pools of soluble and insoluble cytokeratins. However, during necrotic and apoptotic cell death, CK18 and other cytokeratins are released into the blood in either their intact or their caspase-cleaved forms, where they remain relatively stable in the circulation of patients with cancer.^35^ CK18 is proposed as a surrogate measure of disease burden/drug-induced cell death,^36,37^ and it would appear that rising levels in this patient population is indicative of impending disease progression.

Similarly, rising levels of VEGFR2 (in all patients) were associated with a shorter OS. As a target of cediranib, it is not unexpected to observe a fall in levels of circulating VEGFR2, but it is interesting to note that an increase in the levels in all patients is associated with disease progression and suggests a potential mechanism of resistance to chemotherapy.

Whilst we reported that CellSearch-identified CTCs were prognostic at baseline in ABC-03,^17^ the data presented here does not support their role as predictive biomarkers for cediranib. A limitation of these data is that only a subgroup (43 patients for the CTC subgroup) had complete data. Moreover, the CellSearch platform captures only EpCam-expressing CTCs; in many epithelial cancer types, this represents only a subset of CTCs not measuring, for example, the CTCs undergoing epithelial-to-mesenchymal transition. Further studies would be required using marker-independent CTC platforms, which accommodate phenotypic heterogeneity coupled with molecular analysis of DNA profiles of the isolated CTC candidates, to allow detailed evaluation of their utility in the clinical setting. Future studies would also benefit from the collection of the genomic profiling data, which may provide methods for treatment selection.

This translational component to the clinical study was set out to evaluate the biomarkers usefulness, as suggested by others.^38^ These data provide additional information about a panel of circulating biomarkers, which may predict the benefit from the combination of chemotherapy and cediranib.

These data suggest that the treatment with cediranib may attenuate the increased risk of progression and death associated with high circulating levels of VEGFA. It is not known whether this is true for other VEGFA inhibitors such as bevacizumab.

The strength of this study is the prospective evaluation of the sequential biomarker analysis in a randomised cohort of patients against a control, as described in the Cancer Research UK biomarker roadmap (www.cruk.org.uk). We have been able to differentiate between chemotherapy- and cediranib-related effects, and have demonstrated that week 9 (cycle 3) is a suitable time point for the biomarker estimation.

The primary limitation of this study was the necessary “self-selection” of patients for whom the data were available, as only patients who were deemed to have derived clinical benefit (by the absence of disease progression on treatment) contributed longitudinal biomarker data. In addition, this biomarker substudy serves as an exploratory dataset that was not powered a priori to identify the robust subgroups and not adjusted for multiple testing; the findings would need to be validated in an independent dataset, according to the REMARK guidelines.^39^

## Conclusion

Unravelling the complexity of circulating biomarkers is best achieved though prospective randomised trials such as ABC-03. These data propose that the detrimental outcome observed on PFS and OS associated with circulating VEGFA levels in patients with advanced biliary tract cancer treated with cisplatin and gemcitabine may be attenuated by cediranib. This is in keeping with its known mechanism of action. The role of VEGF inhibition requires further evaluation to identify and validate biomarker-defined potentially responsive subgroups. Surrogate measures of tumour burden (rising CK18) and potential treatment resistance (rising VEGFR2) were associated with worse outcomes and warrant validation.

## Electronic supplementary material

Supplementary Figure S1

Supplementary Figure S2A

Supplementary Figure S2B

Supplementary Figure S3

## References

[1] Bragazzi MC (2012). Cholangiocarcinoma: epidemiology and risk factors. Transl. Gastrointest. Cancer.

[2] Bertran E, Heise K, Andia ME, Ferreccio C (2010). Gallbladder cancer: incidence and survival in a high-risk area of Chile. Int. J. Cancer.

[3] Horgan AM, Amir E, Walter T, Knox JJ (2012). Adjuvant therapy in the treatment of biliary tract cancer: a systematic review and meta-analysis. J. Clin. Oncol..

[4] Primrose JN (2017). Adjuvant capecitabine for biliary tract cancer: The BILCAP randomized study. J. Clin. Oncol..

[5] Glimelius B (1996). Chemotherapy improves survival and quality of life in advanced pancreatic and biliary cancer. Ann. Oncol..

[6] Valle J (2010). ABC-02 Trial Investigators. Cisplatin plus gemcitabine versus gemcitabine for biliary tract cancer. N. Engl. J. Med..

[7] Okusaka T (2010). Gemcitabine alone or in combination with cisplatin in patients with biliary tract cancer: a comparative multicentre study in Japan. Br. J. Cancer.

[8] Valle JW (2016). Biliary cancer: ESMO Clinical Practice Guidelines for diagnosis, treatment and follow-up. Ann. Oncol..

[9] Yoshikawa D (2008). Clinicopathological and prognostic significance of EGFR, VEGF, and HER2 expression in cholangiocarcinoma. Br. J. Cancer.

[10] Giatromanolaki A, Koukourakis MI, Simopoulos C, Polychronidis A, Sivridis E (2003). Vascular endothelial growth factor (VEGF) expression in operable gallbladder carcinomas. Eur. J. Surg. Oncol..

[11] Tang D (2006). Angiogenesis in cholangiocellular carcinoma: expression of vascular endothelial growth factor, angiopoietin-1/2, thrombospondin-1 and clinicopathological significance. Oncol. Rep..

[12] Shirabe K (2004). Prognostic factors in node-negative intrahepatic cholangiocarcinoma with special reference to angiogenesis. Am. J. Surg..

[13] Möbius C (2007). Evaluation of VEGF A expression and microvascular density as prognostic factors in extrahepatic cholangiocarcinoma. Eur. J. Surg. Oncol..

[14] Hida Y (1999). Vascular endothelial growth factor expression is an independent negative predictor in extrahepatic biliary tract carcinomas. Anticancer Res..

[15] Benckert C (2003). Transforming growth factor beta 1 stimulates vascular endothelial growth factor gene transcription in human cholangiocellular carcinoma cells. Cancer Res..

[16] Wedge SR (2005). AZD2171: a highly potent, orally bioavailable, vascular endothelial growth factor receptor-2 tyrosine kinase inhibitor for the treatment of cancer. Cancer Res..

[17] Valle JW (2015). Cediranib or placebo in combination with cisplatin and gemcitabine chemotherapy for patients with advanced biliary tract cancer (ABC-03): a randomised phase 2 trial. Lancet Oncol..

[18] Backen AC (2009). ‘Fit-for-purpose’ validation of SearchLight multiplex ELISAs of angiogenesis for clinical trial use. J. Immunol. Methods.

[19] Feldstein AE (2009). Cytokeratin-18 fragment levels as noninvasive biomarker for nonalcoholic steatohepatitis: a multicenter validation study. Hepatology.

[20] Greystoke A (2008). Optimisation of circulating biomarkers of cell death for routine clinical use. Ann. Oncol..

[21] Riethdorf S (2007). Detection of circulating tumor cells in peripheral blood of patients with metastatic breast cancer: a validation study of the CellSearch system. Clin. Cancer Res..

[22] Zhu AX (2013). Efficacy, safety, pharmacokinetics, and biomarkers of cediranib monotherapy in advanced hepatocellular carcinoma: a phase II study. Clin. Cancer Res..

[23] Batchelor TT (2010). Phase II study of cediranib, an oral pan-vascular endothelial growth factor receptor tyrosine kinase inhibitor, in patients with recurrent glioblastoma. J. Clin. Oncol..

[24] Drevs J (2007). Phase I clinical study of AZD2171, an oral vascular endothelial growth factor signaling inhibitor, in patients with advanced solid tumors. J. Clin. Oncol..

[25] Fiedler W (2010). An open-label, Phase I study of cediranib (RECENTIN) in patients with acute myeloid leukemia. Leuk. Res..

[26] Judson I (2014). Phase II study of cediranib in patients with advanced gastrointestinal stromal tumors or soft-tissue sarcoma. Clin. Cancer Res..

[27] Batchelor TT (2013). Phase III randomized trial comparing the efficacy of cediranib as monotherapy, and in combination with lomustine, versus lomustine alone in patients with recurrent glioblastoma. J. Clin. Oncol..

[28] Symonds RP (2015). Cediranib combined with carboplatin and paclitaxel in patients with metastatic or recurrent cervical cancer (CIRCCa): a randomised, double-blind, placebo-controlled phase 2 trial. Lancet Oncol..

[29] van Cruijsen H (2010). Phase I evaluation of cediranib, a selective VEGFR signalling inhibitor, in combination with gefitinib in patients with advanced tumours. Eur. J. Cancer.

[30] Pommier AJ (2014). Serum protein profiling reveals baseline and pharmacodynamic biomarker signatures associated with clinical outcome in mCRC patients treated with chemotherapy ± cediranib. Br. J. Cancer.

[31] Mulders P (2012). Cediranib monotherapy in patients with advanced renal cell carcinoma: results of a randomised phase II study. Eur. J. Cancer.

[32] Hyams DM (2013). Cediranib in combination with fulvestrant in hormone-sensitive metastatic breast cancer: a randomized Phase II study. Invest New Drugs.

[33] Fukuhara S (2008). Differential function of Tie2 at cell-cell contacts and cell-substratum contacts regulated by angiopoietin-1. Nat. Cell Biol..

[34] Huang H, Bhat A, Woodnutt G, Lappe R (2010). Targeting the ANGPT-TIE2 pathway in malignancy. Nat. Rev. Cancer.

[35] Makino T (2009). Cytokeratins 18 and 8 are poor prognostic markers in patients with squamous cell carcinoma of the oesophagus. Br. J. Cancer.

[36] Hägg M (2002). A novel high-through-put assay for screening of pro-apoptotic drugs. Invest. New Drugs.

[37] Bivén K (2003). A novel assay for discovery and characterization of pro-apoptotic drugs and for monitoring apoptosis in patient sera. Apoptosis.

[38] Jain RK, Duda DG, Clark JW, Loeffler JS (2006). Lessons from phase III clinical trials on anti-VEGF therapy for cancer. Nat. Clin. Prac. Oncol..

[39] McShane LM (2005). Statistics Subcommittee of the NCI-EORTC Working Group on Cancer Diagnostics. Reporting recommendations for tumor MARKer prognostic studies (REMARK). Nat. Clin. Pract. Oncol..

