# Supplementary material for: Circulating biomarkers during treatment in patients with advanced biliary tract cancer receiving cediranib in the UK ABC-03 trial
Source: Br J Cancer. 2018 Jun 21;119(1):27–35. doi: 10.1038/s41416-018-0132-8 (PMC6035166; doi:10.1038/s41416-018-0132-8)
Supplement: Supplementary file 1 — Supplementary Figure S1 [file 41416_2018_132_MOESM1_ESM.pptx]

## Slide 1
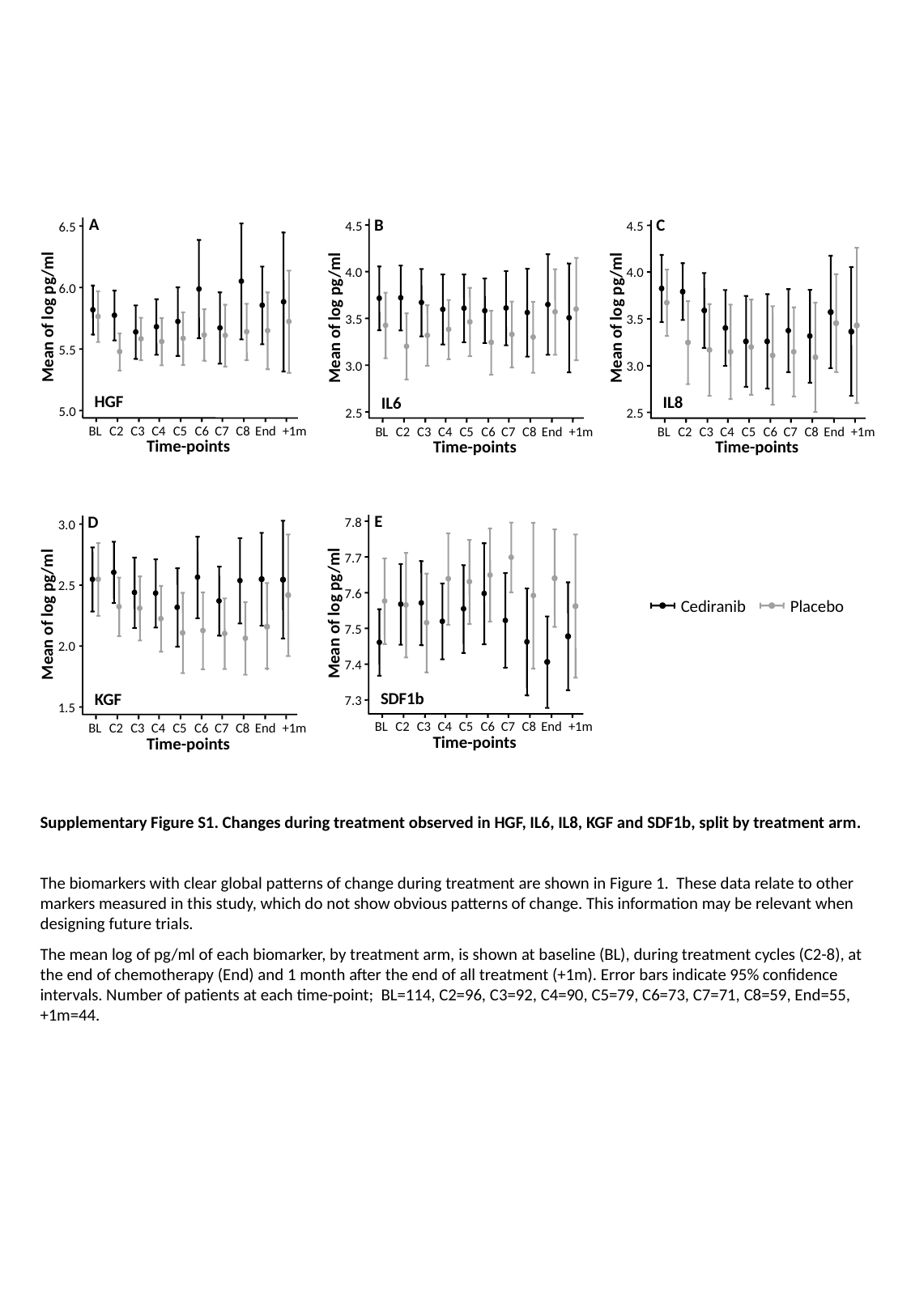

A
6.5
6.0
Mean of log pg/ml
5.5
HGF
5.0
BL
C2
C3
C4
C5
C6
C7
C8
End
+1m
Time-points
B
4.5
4.0
Mean of log pg/ml
3.5
3.0
IL6
2.5
BL
C2
C3
C4
C5
C6
C7
C8
End
+1m
Time-points
C
4.5
4.0
Mean of log pg/ml
3.5
3.0
IL8
2.5
BL
C2
C3
C4
C5
C6
C7
C8
End
+1m
Time-points
E
7.8
7.7
7.6
Mean of log pg/ml
7.5
7.4
SDF1b
7.3
BL
C2
C3
C4
C5
C6
C7
C8
End
+1m
Time-points
D
3.0
2.5
Mean of log pg/ml
2.0
KGF
1.5
BL
C2
C3
C4
C5
C6
C7
C8
End
+1m
Time-points
Cediranib
Placebo
Supplementary Figure S1. Changes during treatment observed in HGF, IL6, IL8, KGF and SDF1b, split by treatment arm.
The biomarkers with clear global patterns of change during treatment are shown in Figure 1. These data relate to other markers measured in this study, which do not show obvious patterns of change. This information may be relevant when designing future trials.
The mean log of pg/ml of each biomarker, by treatment arm, is shown at baseline (BL), during treatment cycles (C2-8), at the end of chemotherapy (End) and 1 month after the end of all treatment (+1m). Error bars indicate 95% confidence intervals. Number of patients at each time-point; BL=114, C2=96, C3=92, C4=90, C5=79, C6=73, C7=71, C8=59, End=55, +1m=44.
