# Supplementary material for: Circulating biomarkers during treatment in patients with advanced biliary tract cancer receiving cediranib in the UK ABC-03 trial
Source: Br J Cancer. 2018 Jun 21;119(1):27–35. doi: 10.1038/s41416-018-0132-8 (PMC6035166; doi:10.1038/s41416-018-0132-8)
Supplement: Supplementary file 2 — Supplementary Figure S2A [file 41416_2018_132_MOESM2_ESM.pptx]

## Slide 1
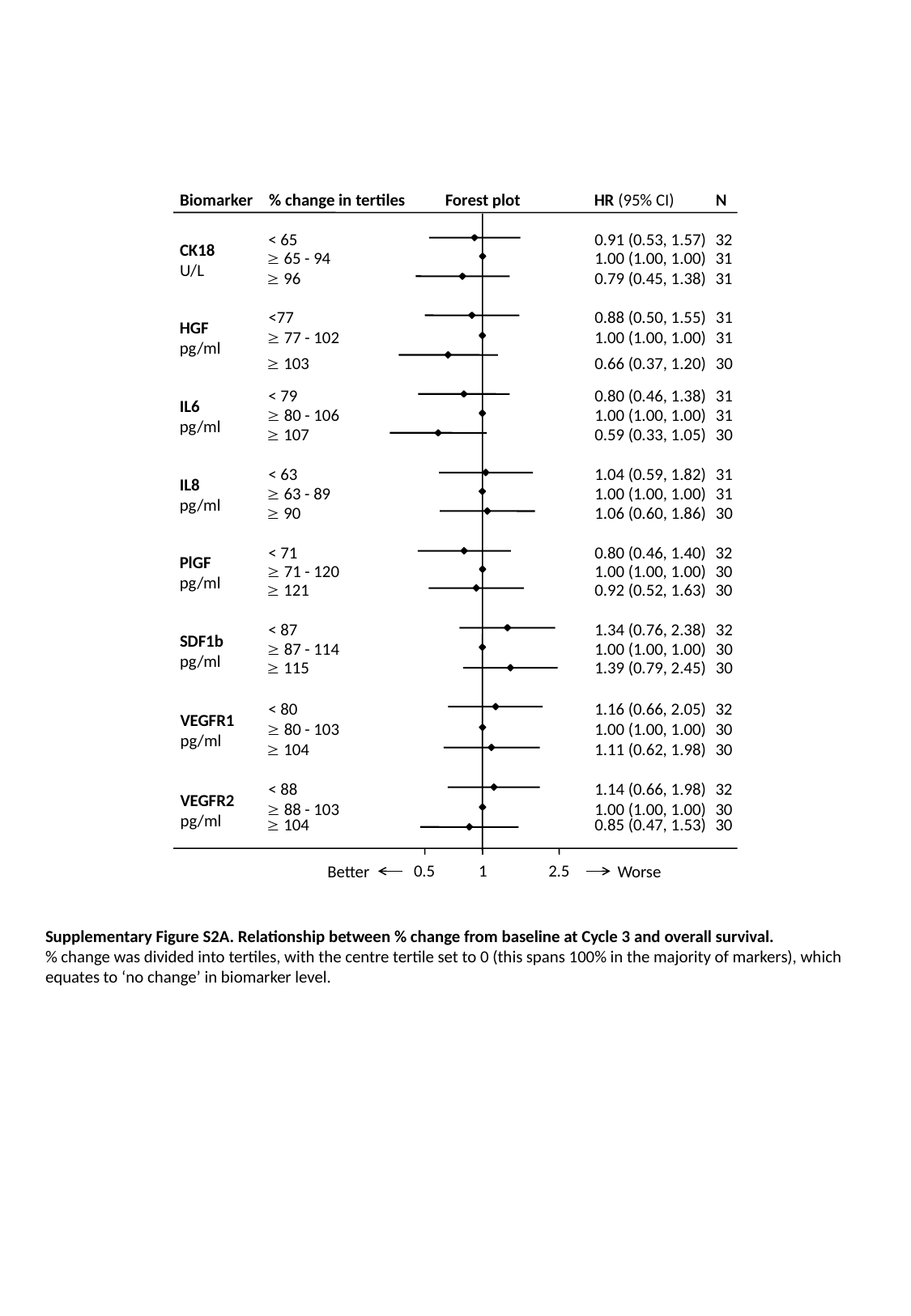

Biomarker
% change in tertiles
Forest plot
HR (95% CI)
N
< 65
0.91 (0.53, 1.57)
32
CK18
U/L
≥ 65 - 94
1.00 (1.00, 1.00)
31
≥ 96
0.79 (0.45, 1.38)
31
<77
0.88 (0.50, 1.55)
31
HGF
pg/ml
≥ 77 - 102
1.00 (1.00, 1.00)
31
≥ 103
0.66 (0.37, 1.20)
30
< 79
0.80 (0.46, 1.38)
31
IL6
pg/ml
≥ 80 - 106
1.00 (1.00, 1.00)
31
≥ 107
0.59 (0.33, 1.05)
30
< 63
1.04 (0.59, 1.82)
31
IL8
pg/ml
≥ 63 - 89
1.00 (1.00, 1.00)
31
≥ 90
1.06 (0.60, 1.86)
30
< 71
0.80 (0.46, 1.40)
32
PlGF
pg/ml
≥ 71 - 120
1.00 (1.00, 1.00)
30
≥ 121
0.92 (0.52, 1.63)
30
< 87
1.34 (0.76, 2.38)
32
SDF1b
pg/ml
≥ 87 - 114
1.00 (1.00, 1.00)
30
≥ 115
1.39 (0.79, 2.45)
30
< 80
1.16 (0.66, 2.05)
32
VEGFR1
pg/ml
≥ 80 - 103
1.00 (1.00, 1.00)
30
≥ 104
1.11 (0.62, 1.98)
30
< 88
1.14 (0.66, 1.98)
32
VEGFR2
pg/ml
≥ 88 - 103
1.00 (1.00, 1.00)
30
≥ 104
0.85 (0.47, 1.53)
30
0.5
1
2.5
Better
Worse
Supplementary Figure S2A. Relationship between % change from baseline at Cycle 3 and overall survival.
% change was divided into tertiles, with the centre tertile set to 0 (this spans 100% in the majority of markers), which equates to ‘no change’ in biomarker level.
