# Supplementary material for: Circulating biomarkers during treatment in patients with advanced biliary tract cancer receiving cediranib in the UK ABC-03 trial
Source: Br J Cancer. 2018 Jun 21;119(1):27–35. doi: 10.1038/s41416-018-0132-8 (PMC6035166; doi:10.1038/s41416-018-0132-8)
Supplement: Supplementary file 3 — Supplementary Figure S2B [file 41416_2018_132_MOESM3_ESM.pptx]

## Slide 1
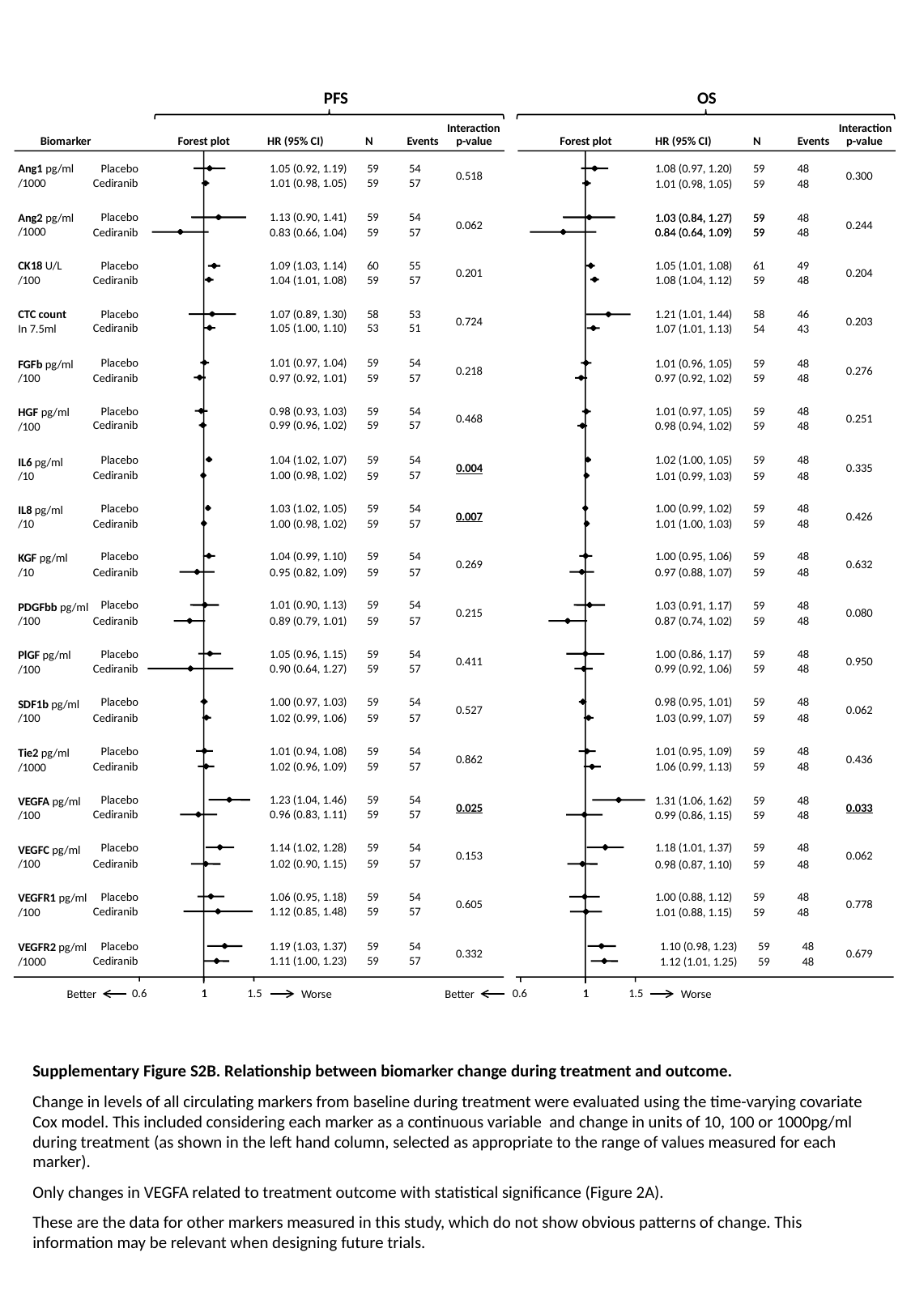

PFS
OS
Interaction
Interaction
Biomarker
Forest plot
HR (95% CI)
N
Events
p-value
Forest plot
HR (95% CI)
N
Events
p-value
Placebo
1.05 (0.92, 1.19)
59
54
1.08 (0.97, 1.20)
59
48
Ang1 pg/ml
/1000
0.518
0.300
Cediranib
1.01 (0.98, 1.05)
59
57
1.01 (0.98, 1.05)
59
48
Placebo
1.13 (0.90, 1.41)
59
54
1.03 (0.84, 1.27)
1.03 (0.84, 1.27)
59
59
48
Ang2 pg/ml
/1000
0.062
0.244
Cediranib
0.83 (0.66, 1.04)
59
57
0.84 (0.64, 1.09)
0.84 (0.64, 1.09)
59
59
48
CK18 U/L
/100
1.05 (1.01, 1.08)
61
49
Placebo
1.09 (1.03, 1.14)
60
55
0.201
0.204
1.08 (1.04, 1.12)
59
48
Cediranib
1.04 (1.01, 1.08)
59
57
Placebo
1.07 (0.89, 1.30)
58
53
CTC count
In 7.5ml
1.21 (1.01, 1.44)
58
46
0.724
0.203
Cediranib
1.05 (1.00, 1.10)
53
51
1.07 (1.01, 1.13)
54
43
Placebo
1.01 (0.97, 1.04)
59
54
1.01 (0.96, 1.05)
59
48
FGFb pg/ml
/100
0.218
0.276
Cediranib
0.97 (0.92, 1.01)
59
57
0.97 (0.92, 1.02)
59
48
Placebo
0.98 (0.93, 1.03)
59
54
1.01 (0.97, 1.05)
59
48
HGF pg/ml
/100
0.468
0.251
Cediranib
0.99 (0.96, 1.02)
59
57
0.98 (0.94, 1.02)
59
48
Placebo
1.04 (1.02, 1.07)
59
54
1.02 (1.00, 1.05)
59
48
IL6 pg/ml
/10
0.004
0.335
Cediranib
1.00 (0.98, 1.02)
57
59
1.01 (0.99, 1.03)
59
48
Placebo
1.03 (1.02, 1.05)
59
54
1.00 (0.99, 1.02)
59
48
IL8 pg/ml
/10
0.007
0.426
Cediranib
1.00 (0.98, 1.02)
59
57
1.01 (1.00, 1.03)
59
48
Placebo
1.04 (0.99, 1.10)
59
54
1.00 (0.95, 1.06)
59
48
KGF pg/ml
/10
0.269
0.632
Cediranib
0.95 (0.82, 1.09)
59
57
0.97 (0.88, 1.07)
59
48
Placebo
1.01 (0.90, 1.13)
59
54
1.03 (0.91, 1.17)
59
48
PDGFbb pg/ml
/100
0.215
0.080
Cediranib
0.89 (0.79, 1.01)
59
57
0.87 (0.74, 1.02)
59
48
Placebo
1.05 (0.96, 1.15)
59
54
1.00 (0.86, 1.17)
59
48
PlGF pg/ml
/100
0.411
0.950
Cediranib
0.90 (0.64, 1.27)
59
57
0.99 (0.92, 1.06)
59
48
Placebo
1.00 (0.97, 1.03)
59
54
0.98 (0.95, 1.01)
59
48
SDF1b pg/ml
/100
0.527
0.062
Cediranib
1.02 (0.99, 1.06)
59
57
1.03 (0.99, 1.07)
59
48
Placebo
1.01 (0.94, 1.08)
59
54
1.01 (0.95, 1.09)
59
48
Tie2 pg/ml
/1000
0.862
0.436
Cediranib
1.02 (0.96, 1.09)
59
57
1.06 (0.99, 1.13)
59
48
Placebo
1.23 (1.04, 1.46)
59
54
1.31 (1.06, 1.62)
59
48
VEGFA pg/ml
/100
0.025
0.033
Cediranib
0.96 (0.83, 1.11)
59
57
0.99 (0.86, 1.15)
59
48
Placebo
1.14 (1.02, 1.28)
59
54
VEGFC pg/ml
/100
Cediranib
1.02 (0.90, 1.15)
59
57
1.18 (1.01, 1.37)
59
48
0.98 (0.87, 1.10)
59
48
0.153
0.062
Placebo
1.06 (0.95, 1.18)
59
54
VEGFR1 pg/ml
/100
Cediranib
1.12 (0.85, 1.48)
59
57
1.00 (0.88, 1.12)
59
48
1.01 (0.88, 1.15)
59
48
0.605
0.778
Placebo
1.19 (1.03, 1.37)
59
54
1.10 (0.98, 1.23)
59
48
VEGFR2 pg/ml
/1000
Cediranib
1.11 (1.00, 1.23)
59
57
1.12 (1.01, 1.25)
59
48
0.332
0.679
0.6
1
1
1.5
0.6
1
1
1.5
Better
Worse
Better
Worse
Supplementary Figure S2B. Relationship between biomarker change during treatment and outcome.
Change in levels of all circulating markers from baseline during treatment were evaluated using the time-varying covariate Cox model. This included considering each marker as a continuous variable and change in units of 10, 100 or 1000pg/ml during treatment (as shown in the left hand column, selected as appropriate to the range of values measured for each marker).
Only changes in VEGFA related to treatment outcome with statistical significance (Figure 2A).
These are the data for other markers measured in this study, which do not show obvious patterns of change. This information may be relevant when designing future trials.
