# Supplementary material for: Circulating biomarkers during treatment in patients with advanced biliary tract cancer receiving cediranib in the UK ABC-03 trial
Source: Br J Cancer. 2018 Jun 21;119(1):27–35. doi: 10.1038/s41416-018-0132-8 (PMC6035166; doi:10.1038/s41416-018-0132-8)
Supplement: Supplementary file 4 — Supplementary Figure S3 [file 41416_2018_132_MOESM4_ESM.pptx]

## Slide 1
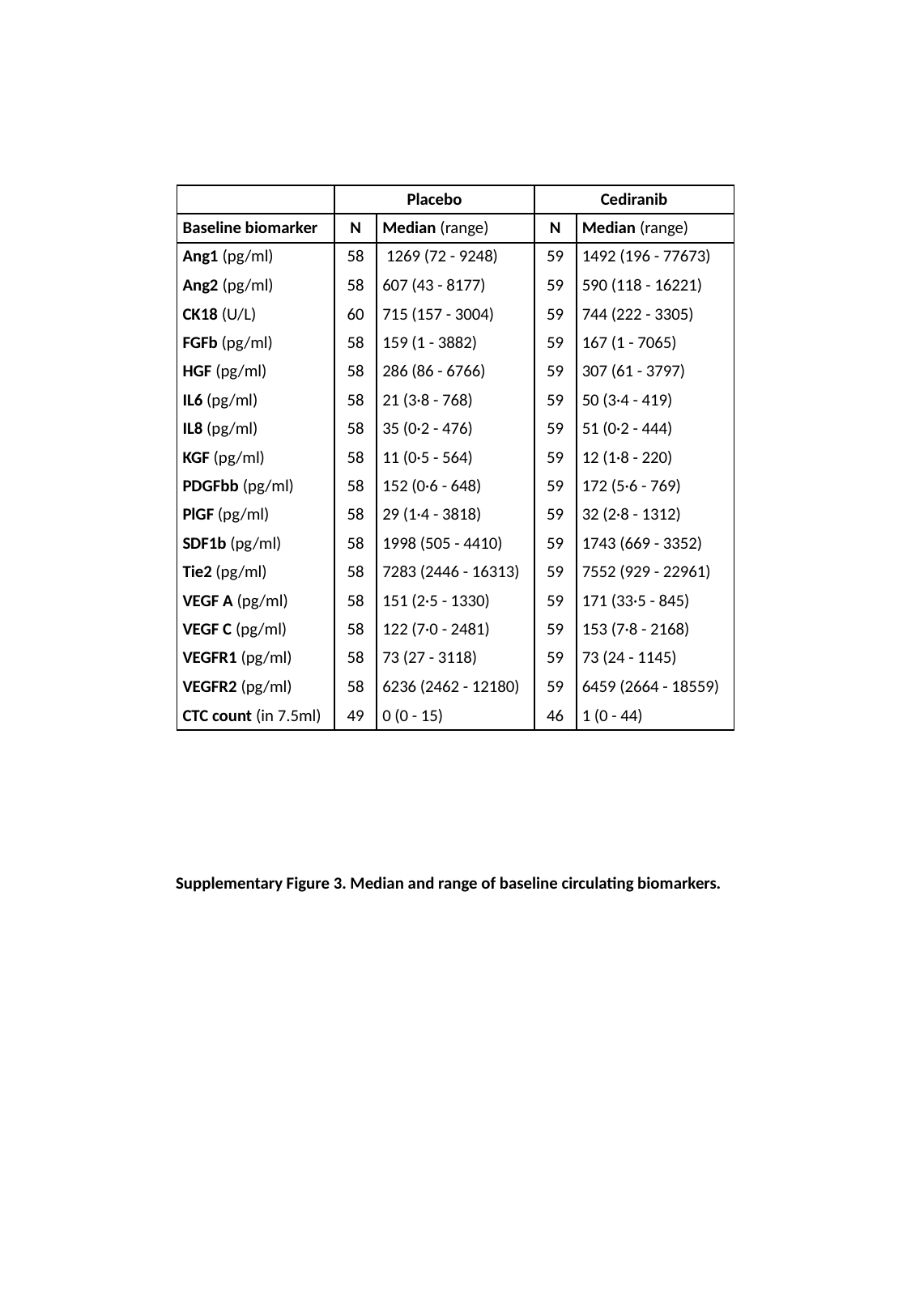

| | Placebo | | Cediranib | |
| --- | --- | --- | --- | --- |
| Baseline biomarker | N | Median (range) | N | Median (range) |
| Ang1 (pg/ml) | 58 | 1269 (72 - 9248) | 59 | 1492 (196 - 77673) |
| Ang2 (pg/ml) | 58 | 607 (43 - 8177) | 59 | 590 (118 - 16221) |
| CK18 (U/L) | 60 | 715 (157 - 3004) | 59 | 744 (222 - 3305) |
| FGFb (pg/ml) | 58 | 159 (1 - 3882) | 59 | 167 (1 - 7065) |
| HGF (pg/ml) | 58 | 286 (86 - 6766) | 59 | 307 (61 - 3797) |
| IL6 (pg/ml) | 58 | 21 (3·8 - 768) | 59 | 50 (3·4 - 419) |
| IL8 (pg/ml) | 58 | 35 (0·2 - 476) | 59 | 51 (0·2 - 444) |
| KGF (pg/ml) | 58 | 11 (0·5 - 564) | 59 | 12 (1·8 - 220) |
| PDGFbb (pg/ml) | 58 | 152 (0·6 - 648) | 59 | 172 (5·6 - 769) |
| PlGF (pg/ml) | 58 | 29 (1·4 - 3818) | 59 | 32 (2·8 - 1312) |
| SDF1b (pg/ml) | 58 | 1998 (505 - 4410) | 59 | 1743 (669 - 3352) |
| Tie2 (pg/ml) | 58 | 7283 (2446 - 16313) | 59 | 7552 (929 - 22961) |
| VEGF A (pg/ml) | 58 | 151 (2·5 - 1330) | 59 | 171 (33·5 - 845) |
| VEGF C (pg/ml) | 58 | 122 (7·0 - 2481) | 59 | 153 (7·8 - 2168) |
| VEGFR1 (pg/ml) | 58 | 73 (27 - 3118) | 59 | 73 (24 - 1145) |
| VEGFR2 (pg/ml) | 58 | 6236 (2462 - 12180) | 59 | 6459 (2664 - 18559) |
| CTC count (in 7.5ml) | 49 | 0 (0 - 15) | 46 | 1 (0 - 44) |
Supplementary Figure 3. Median and range of baseline circulating biomarkers.
